# God save the queen! How and why the dominant evergreen species of the Mediterranean Basin is declining?

**DOI:** 10.1093/aobpla/plad051

**Published:** 2023-08-01

**Authors:** Francesca Alderotti, Erika Verdiani

**Affiliations:** Department of Agriculture, Food, Environment and Forestry (DAGRI), University of Florence, Sesto Fiorentino, Florence 50019, Italy; Department of Agriculture, Food, Environment and Forestry (DAGRI), University of Florence, Sesto Fiorentino, Florence 50019, Italy

**Keywords:** Climate change, dieback, Mediterranean basin, Phytophthora cinnamomi, Quercus ilex decline

## Abstract

*Quercus ilex* may be considered the *queen* tree of the Mediterranean Basin, dominating coastal forest areas up to 2000 m above sea level at some sites. However, an increase in holm oak decline has been observed in the last decade. In this review, we analysed the current literature to answer the following questions: what are the traits that allow holm oak to thrive in the Mediterranean environment, and what are the main factors that are currently weakening this species? In this framework, we attempt to answer these questions by proposing a triangle as a graphical summary. The first vertex focuses on the main morpho-anatomical, biochemical and physiological traits that allow holm oak to dominate Mediterranean forests. The other two vertices consider abiotic and biotic stressors that are closely related to holm oak decline. Here, we discuss the current evidence of holm oak responses to abiotic and biotic stresses and propose a possible solution to its decline through adequate forest management choices, thus allowing the species to maintain its ecological domain.

## Teaser

*Quercus ilex* (holm oak) is one of the most widespread arboreal sclerophylls in Mediterranean forests. Despite its dominance in Mediterranean forests, many dieback episodes have been reported for this species in southern Europe. Holm oak dieback episodes are generally associated with extreme events such as wildfires, heat waves and droughts; however, we hypothesized that an increase in winter drought spells might have contributed significantly to the decline of this species. Among the biotic factors threatening holm oak, root rot induced by *Phytophthora cinnamomi* can directly result in tree mortality. Mitigation practices that control holm oak decline include adaptive silviculture, integrated pest management, chemical control and management of understory vegetation, grazers and browsers. Furthermore, the use of holm oak genotypes tolerant to drought stress and *P. cinnamomi* provides a valuable opportunity to restore declining holm oak forests.

## Introduction

Climate change refers to variations in the mean values and properties of the climate that persist over an extended period, typically decades or longer (Pachauri and Reisinger 2007). Extreme weather events, increasing drought spells and heat waves are causing forest dieback and tree mortality in areas where tree species are not generally subjected to drought stress (tropical environments or boreal forests) and in Mediterranean ecosystems where aridity already limits plant performances (Galmés *et al*. 2007; Reyer *et al*. 2013).

The Mediterranean Basin is characterized by high climatic variability and includes the highest number of Mediterranean-type ecosystems. The peculiarity of Mediterranean climate is the seasonality of temperature and rainfall that generates cold and wet winters, opposed to warm and dry summers (Mitrakos 1980; Walter 1985; Lionello *et al*. 2006).

*Quercus ilex* L. (holm oak) may be considered the *queen* of the Mediterranean Basin and is one of the most widespread arboreal sclerophylls in Mediterranean forests (Ogaya and Peñuelas 2021). This species covers a wide geographical range in the Mediterranean Basin and thrives in both semi-arid and peri-humid habitats (Niinemets 2015; Martín-Sánchez *et al.* 2022). However, prolonged, and intense drought events due to climate change are forcing holm oak phenotypic plasticity to its maximum (Matesanz and Valladares 2014).

**Figure 1.**
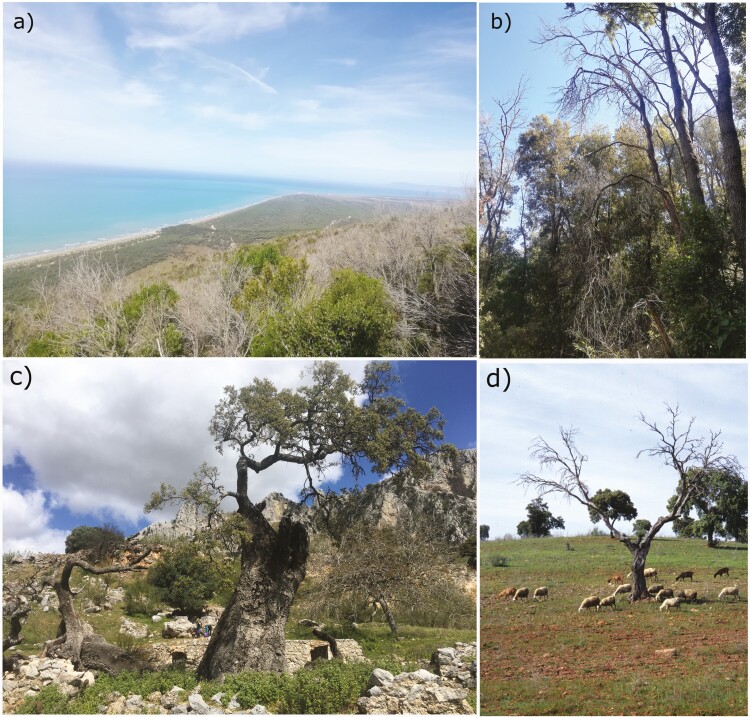
Panoramic (A) and ground view (B) of a declining holm oak forest in a Mediterranean forest stands in Southern Tuscany, Maremma Regional Reserve (Italy). Declining holm oaks in an agro-silvo-pastoral systems (*dehesas*) in Andalusia, Priego de Córdoba, (Southern Spain) (C, D).

Holm oak decline has been mainly reported in Southern Europe (e.g. in the Iberian Peninsula and Italy Fig. 1), and roughly consists of a loss of vigour by trees, identified by

(i) Shoot death and leaf detachment.
(ii) Production of epicormic shoots.
(iii) Fine root loss.
(iv) Decreased growth and increased mortality (Lloret *et al.* 2004a, b; Adams *et al.* 2009; Williams *et al.* 2013; Colangelo *et al.* 2017; Sánchez-Salguero *et al.* 2017a, b).

Forest dieback has been attributed to increased temperatures, reduced soil moisture and increased vapour pressure deficit (Peñuelas *et al*. 2001; Gaylord *et al*. 2013; Ruehr *et al*. 2014). This is often accompanied by attacks by pests and pathogens, including insects, fungi and oomycetes (Boyd *et al.* 2013; Liebhold *et al.* 2017; Jung *et al*. 2018; Contreras-Cornejo *et al.* 2023). Indeed, climate change affects the life cycle and biological synchrony of many forest trees and pathogens, leading to changes in disease impact and distribution (Tubby and Webber 2010; Bosso *et al.* 2016; San-Eufrasio *et al.* 2021a).

**Figure 2.**
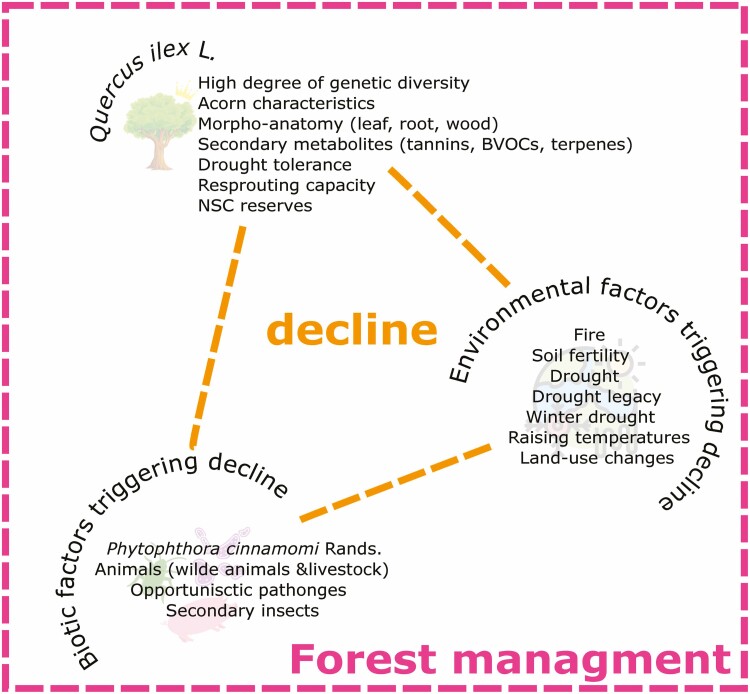
The triangle of *Q. ilex* decline: the frame of the triangle is represented by forest management while the vertices are holm oak, environmental and biotic factors associated with holm oak decline.

In this review, we discuss the characteristics that allow holm oak to thrive in the Mediterranean Basin and the main factors that have weakened this species during the last decades. We try to discuss the phenomenon of holm oak decline by studying the complex network between the abiotic and biotic factors threatening the holm oak domain in the Mediterranean Basin through a triangle figure (Fig. 2).

The first vertex of the triangle focuses on the holm oak and its morpho-anatomical, biochemical, and physiological traits that allow this species to be considered the *queen* of the Mediterranean Basin, at least until today. The other two vertices consider the climatic and biotic factors closely related to holm oak decline. Finally, we propose that adequate forest management choices constitute a possible solution to prevent *Q. ilex* from losing its domain.

## Key Attributes Enabling Holm Oak Dominance in the Mediterranean Basin

*Quercus ilex* is an evergreen broad-leaved sclerophyll species that covers more than 6 million ha in the Mediterranean Basin, mostly in the western region (Ducrey 1992). Since holm oak dominates the Mediterranean landscape, the species was thought to have a European origin; however, recent research strongly supports the East Asian/Himalayan origins of *Quercus* section *ilex* in subtropical-tropical humid forests of the Eocene (Jiang *et al*. 2019).

In Southern Europe, the holm oak presents high population variability, and polymorphism is often associated with a high degree of genetic diversity (Lumaret *et al*. 2002; Valero-Galván *et al*. 2010). The high heterozygosity and allelic richness reported for this species could potentially explain the wide ecological amplitude of the holm oak and its ecophysiological adaptability to water scarcity and thermal stresses (Soto *et al*. 2007; Gimeno *et al*. 2009; Ortego *et al*. 2010; Guzmán *et al*. 2015). Previous studies have associated different holm oak provenances and morphotypes with different tolerances to abiotic (e.g. drought and O_3_) and biotic stresses (Alonso *et al.* 2014; Solla *et al.* 2016; Corcobado *et al.* 2017; San-Eufrasio *et al.* 2020, 2021; Rodríguez-Romero *et al*. 2022a). The high level of DNA variation in evergreen Mediterranean oaks could be due to the local persistence of very ancient genotypes with atavistic characters or hybridization and successive backcrossing that led to the transfer of genes from one species to another (Bellarosa *et al.* 2005; Lopez De Heredia *et al.* 2007, 2017; Burgarella *et al.* 2009). Further, holm oak genetic variability and population structure have been reported even at narrow geographical scale (<20 km), underlining the importance of environmental features (i.e. eco-pedological, climatic, geological) rather than phylogeography in the shaping of holm oak genetic variation and differentiation (Lumaret *et al*. 2002; Vernesi *et al.* 2012).

Holm oak produced recalcitrant seed (i.e. damaged by the loss of water), that despite their sensitivity to water loss, possess a great chance of establishing thanks to their large size, large mass and extremely rich metabolome profile (Quero *et al.* 2007; Sghaier-Hammami *et al.* 2016; Romero-Rodríguez *et al.* 2019).

Regarding the morpho-anatomical features, the holm oak possesses a deep root system that has access to profound soil layers that retain moisture during dry periods (David *et al.* 2007; Padilla *et al.* 2007; Carrière *et al.* 2020). In addition, it has been reported that, during long periods of water stress, this species may lose lateral roots which, in turn, may induce downward root elongation and improve drought tolerance (Chiatante *et al.* 2005). Previous studies exploring the root–shoot ratio revealed a conservative pattern of root mass allocation for holm oak, as in the case of variation in mineral nutrient availability, which preferentially allocates biomass to the root system rather than to the aboveground biomass (Villar-Salvador *et al.*, 2004).

Holm oak has sclerophyll leaves with a dense layer of stellate hairs hiding small and abundant stomata on the lower surface. Marked variation can be observed in leaf characteristics according to their position within the canopy (Terradas and Savé 1992). The long lifespan of holm oak leaves is associated with the high cost of construction necessary to allow leaves to overcome stressful Mediterranean conditions such as intense solar radiation, drought, and low nutrient availability (Montserrat-Martí *et al.* 2009; Sardans and Peñuelas 2013; Alonso-Forn *et al.* 2021). Sclerophylly is a morphological trait traditionally associated with Mediterranean-type climates, with dry and hot summers and frequent salt depo­sition (Mooney and Dunn 1970; Walter 1985; Bussotti *et al.* 2000; Traiser *et al.* 2005). Sclerophyllous species are characterized by high values of leaf density and leaf thickness, both contributing to increase the leaf mass per area (LMA) (Witkowsky and Lamont 1991; Ogaya and Penuelas 2006). However, leaf biochemistry can also increase the LMA. Previous studies have demonstrated a positive relationship between the LMA and leaf tannin content (Gratani *et al.* 2018; Puglielli *et al.* 2019; Alderotti *et al.* 2020).

In general, phenolic compounds, such as tannins, range from 5% to 10% of leaf dry weight (Rossi *et al.* 2004; Barbehenn and Constabel 2011; Grauso *et al*. 2019). The importance of tannins in holm oak leaves was also highlighted by Rodríguez-Romero *et al.* (2022b), who revealed a more stable level of tannins in leaves than in all other organs throughout the year. However, a large variety of secondary metabolites have been identified in holm oak leaves, such as tocopherols, benzenoids, flavonoids and isoprenoids, which play key roles in plant defence against biotic and abiotic stresses (Pasquini *et al.* 2021; Encinas-Valero *et al*. 2022*a*; Tienda-Parrilla *et al.* 2022).

Another class of secondary metabolites produced by holm oak leaves are terpenes, among which monoterpenes are the most abundant (Kesselmeier *et al.* 1997; Simon *et al.* 2005; Pasquini *et al.* 2023). However, holm oak lacks structures for the storage of terpenes, and their emission and production are strongly affected by environmental conditions (Llusià *et al.* 2011). Indeed, terpenes favour plant defence against biotic (herbivores and pathogens) and abiotic stress factors, thus enhancing plant survival under environmental constraints (Copolovici *et al.* 2005; Tienda-Parrilla *et al.* 2022). In general, moderate stress boosts terpene biosynthesis (Staudt *et al.* 2017), while severe stress can greatly reduce their emissions (Loreto and Schnitzler 2010; Niinemets 2010). Indeed, Lavoir *et al.* (2009) found an evident inhibi-tion of holm oak monoterpene emissions in severely water-stressed plants (Ψ_w_ < −2 Mpa). Terpenes are also important during the recovery from drought, as reported by Peñuelas *et al.* (2009), who found that the recovery of monoterpene emissions in water-stressed holm oak seedlings was faster than that of photosynthesis, suggesting a protective role for these compounds. In particular, terpenes may display many protective effects, ranging from antioxidant activity to protection against high temperatures at the cellular level (Loreto *et al*. 2014).

Concerning wood traits, holm oak may adopt xylem anatomical adjustments in response to dry conditions to avoid drought-induced hydraulics disfunctioning (De Micco *et al.* 2007, 2016; Battipaglia *et al.* 2016). Modifications in xylem anatomy (e.g. vessel area and density) have been reported to fluctuate during the growing season in response to envi­ronmental conditions (Corcuera *et al.* 2004; Campelo *et al.* 2010). In particular, wood intra-annual density fluctuations contribute to the plasticity of holm oak xylem (Zalloni *et al.* 2018; Balzano *et al.* 2021). These modifications allow the species to ensure safer control of water transport and better exploitation of water derived from sporadic rain events following periods of summer droughts (Campelo *et al.* 2007; Zalloni *et al.* 2019; Balzano *et al.* 2020). However, few studies have not revealed changes in holm oak xylem structure during dry periods (Limousin *et al.* 2009). Notably, xylem adjustments are induced by climatic conditions occurring only when the cambium is active, which can limit xylem plasticity to sudden extreme climatic events (Martínez-Vilalta *et al.* 2002).

Currently, isohydricity and anisohydricity are reported in the literature as water strategies distinguished based on the extent of water potential variation and stomatal closure to preserve leaf water status on a daily timescale or in water-stressed plants compared to controls. Isohydric plants are thought to be more vulnerable to carbon starvation mortality mechanisms, whereas anisohydric plants are more vulnerable to hydraulic failure (McDowell *et al.* 2008). Holm oak water strategy has been described both as anisohydric (e.g. when compared to Mediterranean *Pinus* spp.) as well as isohydric (e.g. when compared to other co-occurring angiosperms such as *Phillyrea latifolia* L.) (Baquedano and Castillo 2006; Aguadé *et al.* 2015; Trifilò *et al.* 2015; Garcia-Forner *et al.* 2017; Vicente *et al.* 2022). However, despite the difficulty in defining its water strategy, holm oak emerges as a drought-tolerant species, employing a strict stomatal control mechanism to prevent both leaf dehydration and the formation of xylem embolisms (Peguero-Pina *et al.* 2008, 2018; Alonso-Forn *et al.* 2021).

Resprouting is a reproductive strategy in drought-prone ecosystems with high fire frequencies that enables plants to recover immediately after destructive natural damage or management practices (e.g. forest fires, exceptional drought periods, intensive grazing and thinning) (Zeppel *et al*. 2015). Holm oak can resprout owing to its underground reserves in specialized organs (lignotubers) containing concealed buds, non-structural carbohydrates (NSC) (mainly starch), and nutrients that support growth after disturbances (James 1984; Broncano *et al.* 2005; Walters *et al.* 2005; Konstantinidis *et al.* 2006; López *et al.* 2009). Furthermore, unlike basal resprout, post-fire and post-drought epicormic resprouting allows retention of the arborescent skeleton, ensuring quick recovery after fire/drought stress (Pausas and Keeley 2017). Holm oak has shown full canopy recovery within a year after an extreme drought that induced extensive branch desiccation (Ogaya *et al.* 2014; Liu *et al.* 2015). Moreover, the resprouted leaves showed a higher tolerance to severe and moderate drought in terms of gas exchange performances, water relations and photosystem integrity (Peña-Rojas *et al.* 2004). Thus, carbon reserves play a key role in holm oak recovery from disturbance (and, consequently, in its resilience). Indeed, carbon reserve depletion has been associated with deterioration of crown conditions in earlier studies (Bréda *et al.* 2006; Galiano *et al.* 2012; Rosas *et al.* 2013). However, plants that have already resprouted could be more vulnerable to disturbance and dieback phenomena due to temporary depletion of carbohydrate reserves (Díaz-Delgado *et al.* 2002).

## Threats to the Holm Oak Dominance

Within our conceptual framework, we have examined the morpho-anatomical, biochemical, and physiological characteristics that have enabled the holm oak to establish its dominance in the Mediterranean Basin to date. However, several factors such as increased infestation by *P. cinnamomi*, intensified occurrence of extreme climatic events (such as heat waves and droughts), and reductions in precipitation associated with climate change are likely to undermine the holm oak’s domain. Notably, recent assessments in Italy, Portugal, and Spain have elevated the holm oak’s status to threatened, with its conditions deemed unfavourable or inadequate (U1) in accordance with the Habitats Directive—Article 17 (https://www.eionet.europa.eu/article17/habitat/summary/?period=5&group=Forests&subject=9340&region=/). Consequently, given the observed instances of holm oak dieback, the species has been classified as moderately tolerant to mild drought (Limousin *et al.* 2022). In light of this, the question arises: What explains the progressive loss of resilience in holm oak?

### Environmental factors associated with the holm oak decline

Drought and fire are two of the main environmental hazards threatening holm oak health and the Mediterranean forests ecosystem functioning. Despite the reduction in the total annual burned area in Mediterranean Europe during the period 1985–2011 (Turco *et al*. 2016; Urbieta *et al.* 2019), an increase in fire season (March–September period in Europe) severity has been observed (Venäläinen *et al*. 2014). Even with short fire exposure periods, crowns, stumps and roots can be severely damaged (Bond and Van Wilgen 2012; Chiatante *et al*. 2015). Furthermore, wildfires affect soil fertility which is already low in Mediterranean forests (Sardans and Peñuelas 2013; Hinojosa *et al*. 2021). It is worth noting that wildfire ignition and spread are more challenging in agro-silvo-pastoral ecosystems, such as *dehesas* and *montados*, compared to dense holm oak forests. This is primarily due to the lower forest biomass productivity, as well as the reduced fuel and stem density in these ecosystems, typically characterized by approximately 20–40 trees per hectare. Furthermore, the presence of cattle, sheep, and pigs plays a crucial role in controlling shrubs and herbs while also contributing to soil fertilization. However, it is important to note that livestock activities can have adverse effects, such as soil compaction and the accumulation of urea (Brasier 1996; Pinto-Correia and Mascarenhas 1999; Ortega *et al.* 2012; Rolo *et al.* 2012; López-Sánchez *et al.* 2021).

Drought strongly reduces holm oak carbon uptake due to stomatal closure and plants must rely on their own and finite resources to sustain metabolism (Peguero-Pina *et al.* 2008; Galle *et al*. 2011; Rivas-Ubach *et al*. 2014; Forner *et al*. 2020). Hence, the likelihood of experiencing losses in drought resilience significantly increases when forests are subjected to prolonged and recurrent stress with limited recovery periods (Magno *et al*. 2018; Senf *et al*. 2020).

Hydraulic failure may occur in cases of intense droughts that exceed the xylem resistance to embolism of the species. In particular, the hydraulic vulnerability of holm oak was previously linked to its relatively high cuticular conductance which leads to water losses even when stomata are close (Garcia-Forner *et al.* 2017; Peguero-Pina *et al.* 2018). However, the hydraulic threshold for embolism formation in holm oak is still ambiguous because the sampling procedure for this species is particularly complex due to its long-xylem vessels (Cochard and Tyree 1990; Wheeler *et al.* 2013; Torres-Ruiz *et al.* 2015). In addition, it is important to mention that hydraulic conductivity loss may also be accompanied by carbon reserve depletion (Sala *et al.* 2012; Gori *et al.* 2023). Resco de Dios *et al.* (2020) assigned to holm oak’s stored carbon an important role in recovery from drought, however, NSC depletion resulted to limit resprouting only when co-occurring with hydraulic dysfunction. In contrast, a study by Galiano *et al.* (2012) revealed that holm oak experienced depletions in carbohydrate reserves even seven years after a drought event, highlighting the extended duration required for carbon reserves replenishment.

Under stressful environmental conditions, holm oak preferentially allocates carbohydrates to root branching rather than to foliage maintenance (Encinas-Valero *et al*. 2022b). Encinas-Valero *et al.* (2022b) hypothesized that there is a trade-off between root phenotype plasticity and crown foliage, which may result in a negative feedback loop, leading to tree death. Furthermore, defoliation may result from the failure of leaves to counteract oxidative stress through photoprotective mechanisms, leading to a reduction in the photosynthetic surface and a reduction in carbon uptake, which could enable holm oaks to meet the demands of metabolism and growth (Encinas-Valero *et al*. 2022a). In this regard, Heres *et al.* (2018) highlighted the potential role of crown vigour in secondary growth, detecting chronic lower growth in defoliated holm oaks compared to low-defoliated neighbour trees. These are clear examples of the so-called ‘drought legacy effects’; where drought conditions may continue to negatively affect vegetation although they are alleviated (Kannenberg *et al.* 2020). These effects are usually attributed to ecophysiological memory, although the frequency of drought events and the overlapping recovery periods between different episodes of drought could also contribute to such legacies (Szejner *et al.* 2020). However, trees may also suffer from land use legacy, as shown by Gea-Izquierdo *et al*. (2021) in declining Spanish *dehesas* obtained from the conversion of a closed forest to agro-silvo-pastoral use. Nevertheless, slow or a retarded response to a stress agent could both indicate continued impairment or acclimation (Gessler *et al.* 2020). Indeed, plants can acclimate to persistent changes in the environment, preventing long-term impairment of plant function from adaptation to a new equilibrium, thus predicting the fate of holm oak in the long term is very complex.

Notably, temperature increments induced by climate change could reduce the temperature limitation on winter photosynthesis and evergreen oaks may take advantage of the recovery of carbon reserves (Crescente *et al.* 2002; Gea-Izquierdo *et al.*2011). Previous studies have reported positive photosynthetic activity in winter, comparable to that of spring and autumn seasons, in various Mediterranean species, including the holm oak (Gulías *et al.* 2009). This was linked to the downregulation of summer photosynthesis and the higher sensitivity of the photosynthetic system in early autumn, as revealed by Vaz *et al.* (2010), who showed a recovery of the maximum carboxylation rate and the light-saturated rate of photosynthetic electron transport in evergreen Mediterranean oaks after the first autumnal rain events. However, the beneficial effect of temperature increases on winter gas exchanges could be counteracted by winter dry spells (Hacke and Sperry 2001). Indeed, in drought-prone ecosystems, winter groundwater recharge is fundamental for meeting the high summer demand. Therefore, winter dryness can significantly affect the resilience of the Mediterranean forests (Rodriguez-Puebla *et al.* 2007; Pumo *et al.* 2008). Climate model simulations forecast an increase in the frequency, persistence, and extension of very long dry spells in winter over the Mediterranean Basin (Raymond *et al.* 2019). From the period 1957-2013, Raymond *et al.* (2016) detected seventy-six very long dry spells over the Mediterranean basin during the wet season. Furthermore, Brunetti *et al.* (2002) reported an increase in drought conditions in Italy during winter, which was particularly evident in southern regions (Brunetti *et al.* 2012; Caloiero *et al.* 2015). Despite the large number of studies on drought and heat stress in holm oaks (Sperlich *et al*. 2019; Peguero-Pina *et al.* 2020; Martín-Sánchez *et al.* 2022; Gori *et al.* 2023), as far as we know, there is a limited number of studies dealing with the impact of winter drought on holm oak ecophysiology (Nardini *et al.* 2000). Besides, dry winters negatively affect gross and net primary production in evergreen oak species (including *Q. ilex*), increasing the risk of embolism formation due to freeze-thaw events and drought (Allard *et al.* 2008; Costa-e-Silva *et al.* 2015; Forner *et al.* 2018). When water freezes, air comes out of the solution, but it should redissolve in the water when the ice melts; however, in the case of only a small xylem tension, bubbles would expand, determining xylem dysfunction due to embolism (Tyree and Cochard 1996).

### Biotic factors associated with holm oak decline

The increased dieback of holm oak in Mediterranean forests has also been associated with the presence of pests and pathogens (Peñuelas and Sardans 2021). The soilborne pathogen *Phytophthora cinnamomi* is considered one of the main drivers of holm oak decline in Europe, especially in Portugal, Spain, Southern France and Southern Italy (De Sampaio *et al.* 2013; Corcobado *et al.* 2013, 2015; Linaldeddu *et al.* 2010, 2014; Jung *et al.* 2016, 2018; Fernandez-Habas *et al.* 2019).

*Phytophthora cinnamomi*, is a polyphagous pathogen able to grow saprophytically on dead organic matter as well as parasitically on a huge range of susceptible hosts (Hardham and Blackman 2018; Vitale *et al.* 2019). *Phytophthora cinnamomi* is a root pathogen which caused necrosis, cankers, losses of fine and lateral roots. In some cases, the infection can develop up to the collar causing lesioned cankers, often with black exudates (Redondo *et al*. 2015). Pathogen infection interferes with plant water uptake and transport, thus leading to wilting, chlorosis and defoliation. However, plants can die quickly or survive without showing disease symptoms for many years (Denman *et al*. 2009; Hardham and Blackman 2018; Jung *et al.* 2018).

Despite the dry summers of the Mediterranean ecosystem, relatively warm and humid winter and spring conditions are ideal for this pathogen (De Sampaio *et al.* 2013). Additionally, *P. cinnamomi* infection during the rainy season makes plants even more vulnerable to drought-induced mortality, because of their already compromised root and vascular systems (Corcobado *et al.* 2014; Burgess *et al.* 2017).

Recent studies have highlighted the occurrence of several previously unrecovered *Phytophthora* species that inhabit declining holm oak forests, suggesting their involvement in these declining events (Corcobado *et al.* 2010; Pérez-Sierra *et al.* 2013; Scanu *et al.* 2015). The high diversity of *Phytophthora* species in the soil of declining trees has also been supported by metagenomic approaches based on high-throughput sequencing (Ruiz-Gómez *et al.* 2019; Català *et al.* 2017; Mora-Sala *et al.* 2019). The presence of multiple *Phytophthora* species (i.e. *P*. *gonapodyides*, *P*. *quercina* and *P*. *cinnamomi*) on the same site or even on the same tree can result in a more rapid decline of holm oak forests (Corcobado *et al.* 2017). Therefore, it would be important to study the potential interactions among different *Phytophthora* species that affect the same individuals.

In the last 60 years, an increase in the spread of *Phytophthora* spp. has been reported in European Mediterranean forests, and a further increase is expected in the next decades because of the predicted warmer and drier conditions and more frequent extreme climatic events of drought and waterlogging (Lindner *et al*. 2010; Contreras-Cornejo *et al.* 2023). Recent studies have identified *Phytophthor*a spp. and drought as the main cause of oak death in southwest Spain, however, even more intense, an unprecedented holm oak mortality is expected in infected soils areas subjected to drought-flood alternation stress (Marcais *et al.* 2004; Moralejo *et al.* 2009; Corcobado *et al.* 2014; Gallardo *et al.* 2019). However, it is difficult to identify the precise cause of holm oak forest decline, as it is challenging to distinguish between the impacts of drought, increased temperature, and *P. cinnamomi* infestation. This is because *P. cinnamomi* infestation can trigger biochemical defenses and metabolomic shifts that are similar to those induced by drought (Sena *et al.* 2018; Domínguez‐Begines *et al.* 2020; San-Eufrasio *et al.* 2021a).

In addition, *P. cinnamomi* infection may reduce holm oak natural regeneration, further complicating Mediterranean forests and agro-silvo-pastoral system conservation, which are already degraded due to inadequate management practices (Pérez-Sierra *et al.* 2013; Štraus *et al.* 2023). In these areas, a decrease in acorns availability for holm oak’s natural regeneration can also be observed due to the presence of wild animals and livestock feeding activities. This highlights the existence of conflicting forces that select acorns for the offspring generation of holm oak (Gómez 2004). In addition, seedling survival rate and plant architecture may also be altered by animal feeding and overgrazing (in the case of *dehesas*) (Gea-Izquierdo *et al.* 2006; Pausas *et al.* 2009a, b; López-Sánchez *et al*. 2021).

It is worth to note that *Phytophthora* infections can pave the way for other opportunistic pathogens (e.g. fungus of the genus *Armillaria, Diplodia corticola and Biscognauxia mediterranea*) and attack of secondary insects (e.g. *Lymantria dispar*) (Linaldeddu *et al.* 2014; Milanovic *et al.* 2015; Keča *et al.* 2016; Jung *et al.* 2018). Indeed, holm oak decline may also involve contributing factors such as secondary subcortical insect pests (e.g. *Scolytus* spp. (Curculionidae), *Xylotrechus* spp. (Cerambycidae) or *Agrilus* spp. (Buprestidae)) whose attacks on already weakened plants are usually difficult to prevent (Macháčová *et al.* 2022).

Root rot disease has been associated not only with *Phytophthora* infection but also with *Armillaria* spp., an opportunistic pathogen probably contributing to holm oak decline (Luisi *et al.* 1996; Marçais and Bréda 2006).

## Effective Forest Management Strategies for the Conservation of Holm Oak Dominance

In recent decades, disruptive events, such as disease, drought and fire, have forced the adoption of forest management practices aimed at facilitating successional processes and increasing water availability (Troendle *et al*. 2001; Ganatsios *et al*. 2010; Doblas-Miranda *et al*. 2017; Del Campo *et al*. 2019a). Particularly, unmanaged high-density forests with low surface biomass, such as abandoned oak coppices, are prone to climate-related disturbances, underscoring the need to define adaptive treatments to increase oak coppice resilience (Sturrock *et al*. 2011).

Adaptive silviculture methods aimed at regulating competition and the derived effects of density facilitate the functional diversity of forest communities and promote their complexity (Aquilué *et al.* 2021; Borghetti *et al*. 2021). One of the most common practices of adaptive silviculture is selective thinning, which consists of reduction of stem density (Chang *et al*. 2016) to improve forest health and productivity by increasing the solar radiation reaching soil, soil organic matter, water and nutrient availability for the remaining trees (Tang *et al*. 2005; Roberts and Harrington 2008; Selig *et al*. 2008; Sullivan and Sullivan 2016). Selective thinning may alleviate holm oak water stress, especially summer water stress, extending the growing season and increasing stem growth rate as shown by Cabon *et al*. (2018). Thus, selective thinning may have a beneficial effect on stress response and restoration time, especially in mixed forests (Jones *et al.* 2019). Mediterranean coppices thinned with the removal of 30% of holm oak basal area had successfully reduced the mortality rate of this species at an experimental site of rainfall exclusion in the long term (Gavinet *et al*. 2019). However, many drought-resistant shrub species could take advantage of holm oak mortality or basal area reduction highlighting the need for accurate management of undergrowth shrubs, whose cover reduction can result in a higher water availability for trees, thus improving the holm oak conservation (Ogaya and Peñuelas 2003; Barbeta *et al*. 2013; Cabon *et al*. 2018; Del Campo *et al.* 2019a, b; Moreno-Fernández *et al.* 2019).

Although selective thinning may be a valid management solution for dense and declining forests, this choice seems less valuable to control holm oak decline, in *dehesas* or *montados* of Iberia Peninsula, where due to the low density the plants do not compete for resources acquisition (Pulido *et al.* 2014). By contrast, tree isolation of *dehesas*, together with increased mechanization, increased loading rates and changes in grazing practices, contribute to holm oak dieback concurrently with difficulties of tree natural regeneration, dispersal and post-dispersal survival rates (Alejano *et al.* 2008; Moreno and Pulido 2009; Pulido *et al.*, 2010; Carmona *et al.*, 2013). Therefore, some authors have concluded that the recovery of transhumant-based seasonal grazing regimes can help improve *dehesas* conservation status and natural oak regeneration by alleviating the impact of grazers and browsers (Cierjacks & Hensen, 2004; Ramírez and Díaz 2008; Carmona *et al.*, 2013; Leal *et al.* 2022).

Practices aimed at controlling *Phythoptora* spp. include encouraging soil drainage, lime fertilization, the use of biofumigant crops, the elimination of alternative host herbaceous species, and the avoidance and soil movements (Serrano *et al.* 2012; Rios *et al.* 2017; San-Eufrasio *et al.* 2021b). In addition to integrated control, chemical control can be used to mitigate root rot disease, although its applicability may change depending on forest type. Chemical control of *P. cinnamomi* infections generally relies on the use of resistance inducers such as potassium phosphite (K_2_HPO_3_) or fosetyl-aluminium (aluminium tris-O-ethyl phosphonate, fos-al) that reduce disease by implementing the host plant’s natural defence mechanisms to arrest pathogen development (Berkowitz *et al.* 2013). Resistance inducers can be applied at the individual tree level, either through trunk inception or trunk spray or on a larger scale via leaf spray (San-Eufrasio *et al*. 2021b; Solla *et al.* 2021). However, caution should be used when applying these chemical products on a large scale. Previous field studies conducted on holm oak trees infected by *P. cinnamomi* showed that the most promising results were obtained through individual tree trunk injection of trees not stressed by drought (Romero *et al.* 2019; San-Eufrasio *et al.* 2021b).

The use of *P. cinnamomi* -tolerant genotypes provides an alternative to chemical control of the disease. Long-term conventional breeding programs aimed at producing *P. cinnamomi*-tolerant genotypes have not yet been conducted (Martínez *et al.* 2020), *P. cinnamomi-*tolerant genotypes may be vegetatively propagated from surviving adult trees in declining oaks through micropropagation techniques (i.e. axillary shoot proliferation and somatic embryogenesis), despite the difficulties of clonal propagation of oaks (Martínez *et al.* 2020). Nevertheless, the restoration of *P. cinnamomi*-affected areas using tolerant holm oak plant material has greater applicability, as previous greenhouse and field experiments have highlighted that *P. cinnamomi* tolerance can vary according to plant provenance and plant constitutive defences (Corcobado *et al.* 2017; Rodríguez-Romero *et al.* 2022a; 2022b). Furthermore, Vivas *et al.* (2021) found that the offspring of non-infected trees have a higher mortality rate than those of infected trees. Thus, the transgenerational effects of *P. cinnamomi* infection on *Q. ilex* progeny provide opportunities for the long-term natural recovery of holm oaks.

Furthermore, proteomic approaches have addressed various aspects of holm oak resistance to both biotic and abiotic stresses. Therefore, the inclusion of drought-tolerant provenances, in addition to *P. cinnamomi*-tolerant genotypes, should be considered when selecting seed-bearing plants for the conservation of holm oak forests in agro-silvo-pastoral settings (Gimeno *et al.* 2009; Valero-Galvan *et al.* 2013; San-Eufrasio *et al.* 2021a).

## Conclusion

Despite the morpho-anatomical, biochemical, and physiological traits that allow the holm oak to dominate the Mediterranean basin, many dieback episodes have been reported for this species in southern Europe. Extreme events such as wildfires, heat waves and droughts, pose a serious threat to holm oak domain and have been reported to cause holm oak dieback through both carbon starvation and massive xylem hydraulic dysfunction. However, the progressive loss of resilience revealed for holm oak might have to deal with the timing of drought events. Because holm oak is adapted to summer heat and drought stress, we hypothesized that an increase in winter drought spells might have contributed significantly to the loss of resilience of this species. In this context, despite the high number of studies dealing with drought and heat stress in holm oak, there are a limited number of studies on the impact of winter drought on its physiology, which deserves further investigation.

Among the biotic factors threatening holm oak, root rot induced by *P. cinnamomi* can directly result in tree mortality when the infection is sufficiently high. Furthermore, the possibility of multiple *Phytophthora* species living on the same site or plant, difficulties in the detection of early stages of the disease, and the increase in the spread of *Phytophthora species* expected in the next decades, make *Phytophthora* management very difficult. However, in the case of non-lethal infections, *P. cinnamomi* can make holm oak even more vulnerable to drought-induced mortality or pave the way for other opportunistic pathogens and attacks by secondary insects involved in the decline phenomenon.

Mitigation practices that control holm oak decline include adaptive silviculture, integrated pest management and chemical control. Furthermore, the use of holm oak genotypes tolerant to drought stress and *P. cinnamomi* provides a valuable opportunity to restore declining holm oak forests. Finally, accurate management of understory vegetation, grazers and browsers would improve natural oak regeneration thus improving holm oak forests and agro-silvo-pastoral forest conservation.

## Data Availability

No new data were generated for this article.

## Notes

**Natural History and Conservation. Chief Editor:** F. Xavier PicPicó
